# Incidence and severity of immune-related hepatitis after dual checkpoint therapy is linked to younger age independent of herpes virus immunity

**DOI:** 10.1186/s12967-022-03755-3

**Published:** 2022-12-12

**Authors:** Zhen Zhang, David Rafei-Shamsabadi, Saskia Lehr, Nico Buettner, Rebecca Diehl, Daniela Huzly, David J Pinato, Robert Thimme, Frank Meiss, Bertram Bengsch

**Affiliations:** 1 Faculty of Medicine, Clinic for Internal Medicine II, Gastroenterology, Hepatology, Endocrinology, and Infectious Disease, University Medical Center Freiburg, Freiburg, Germany; 2 Faculty of Medicine, Department of Dermatology and Venereology, University Medical Center Freiburg, Freiburg, Germany; 3 Institute of Virology, Faculty of Medicine, Freiburg University Medical Center, University of Freiburg, Freiburg, Germany; 4 Department of Surgery and Cancer, Imperial College London, Hammersmith Hospital, London, UK; 5 Department of Translational Medicine, University of Piemonte Orientale, Novara, Italy; 6 Signalling Research Centres BIOSS and CIBSS, University of Freiburg, Freiburg, Germany; 7 Partner Site Freiburg, German Cancer Consortium (DKTK), Heidelberg, Germany

**Keywords:** Checkpoint therapy, Immune-related adverse effects, Hepatitis, Age, Herpes virus

## Abstract

**Background and Aims:**

Dual immune checkpoint blockade (ICB) therapy can result in immune-related-adverse events (irAE) such as ICB-hepatitis. An expansion of effector-memory (TEM) CD4 T cells associated with antiviral immunity against *herpesviridae* was implicated in ICB-hepatitis. Notably, these memory subsets are frequently associated with age. Here, we sought to understand baseline patient, immune and viral biomarkers associated with the development of ICB-hepatitis to identify currently lacking baseline predictors and test if an expansion of TEM or positive serology against *herpesviridae* can predict ICB-hepatitis.

**Methods:**

A discovery (n = 39) and validation cohort (n = 67) of patients with advanced melanoma undergoing anti-PD-1&anti-CTLA4 combination therapy (total n = 106) were analyzed for baseline clinical characteristics, occurrence of irAE and oncological outcomes alongside serological status for CMV, EBV and HSV. Immune populations were profiled by high-parametric flow cytometry (n = 29).

**Results:**

ICB-hepatitis occurred in 59% of patients within 100 days; 35.9% developed severe (CTCAE 3–4) hepatitis. Incidence of ICB-hepatitis was higher in the younger (< 55y: 85.7%) compared to older (> = 55y: 27.8%) age group (p = 0.0003), occured earlier in younger patients (p < 0.0001). The association of younger age with ICB-Hepatitis was also observed in the validation cohort (p = 0.0486). Incidence of ICB-hepatitis was also associated with additional non-hepatic irAE (p = 0.018), but neither positive IgG serostatus for CMV, EBV or HSV nor TEM subsets despite an association of T cell subsets with age.

**Conclusion:**

Younger age more accurately predicts ICB-hepatitis after anti-PD-1&anti-CTLA4 checkpoint therapy at baseline compared to herpes virus serology or TEM subsets. Younger patients should be carefully monitored for the development of ICB-hepatitis.

**Supplementary Information:**

The online version contains supplementary material available at 10.1186/s12967-022-03755-3.

## Background

Combination immune checkpoint blockade (ICB) immunotherapy with anti-PD-1 & anti-CTLA4 is now in widespread use for unresectable/metastatic melanoma, non-small cell lung cancer (NSCLC) with TPS ≥ 1%, pleural mesothelioma and is currently under intensive evaluation in other oncological indication. The high clinical efficacy of combination immunotherapy however comes at the cost of a higher incidence of immune-related adverse events (irAE). In clinical trials for malignant melanoma, 30–55% patients treated with combinational therapy had severe CTCAE grade 3–4 adverse events [1–5], which limits continuation of therapy, and, in some cases, may lead to significant harm and death [6]. Hepatitis is one of the most common irAE causing severe (CTCAE 3–4) toxicity in anti-PD-1 & anti-CTLA4 therapy with incidence rates reported up to 33% [1, 3, 7]. Immune-related hepatitis (ICB-hepatitis) is diagnosed during checkpoint blockade therapy based on changes in Alanine-Aminotransferase (ALT), Aspartate-Aminotransferase (AST) and other indices of liver function following exclusion of alternative etiologies of hepatitis [8]. Management strategies range from close observation to immunosuppressive therapy depending on CTCAE grading [9].

However, despite emerging evidence of dynamic changes in immune cell function in ICB-hepatitis [10], there is currently a lack of precise mechanistic understanding of the pathogenesis of this new disease entity, leading to the lack of effective prophylactic management and patient-tailored surveillance strategies. Recently, a potential association between baseline immune responses and the occurrence of severe irAE and ICB-hepatitis was reported. Lozano et al. described a link between activated CD4+ effector memory T cell (T_EM_) populations and the development of severe adverse events after anti-PD-1/combinational blockade therapy [11]. Hutchinson et al. reported an enrichment of CMV-associated T_EM_ CD4 populations in the peripheral blood of patients who further developed hepatitis in their cohort [12], instigating a provocative suggestion whether introduction of selective antivirals against *Herpesviridae* might be beneficial in the prevention or therapy of checkpoint-related immune hepatitis.

Exposure to *Herpesviridae* as evidenced by seroprevalence against CMV, EBV or HSV 1–2 is increasing with age [13]. However, many changes in T cell populations, such as the reduction of naïve T cells and accumulation of T_EM_ or T_EMRA_ cells are associated with aging, and age together with CMV infection have been identified as major variables associated with expansion of TEM cells, including in cohorts of monocygotic twins [14, 15]. Thus, age and CMV infection may both contribute to expansion of T_EM_ CD4 responses and affect the incidence ICB-hepatitis. In this study we therefore sought to understand the role of age, gender and baseline herpes virus immunity in a prospectively recruited discovery and retrospective validation cohort of stage III/IV melanoma patients treated with anti-PD-1 & anti-CTLA4 combination therapy reflecting real-world patient cohorts at a tertiary academic medical center.

Our data from n = 106 stage III/IV melanoma patients who received combinational ICB therapy with anti-PD-1 and anti-CTLA4 identifies age, but not underlying herpes virus immunity or peripheral TEM subsets as the major variable associated with the risk for immune-checkpoint associated hepatitis.

## Methods

### Patient recruitment

Melanoma patients treated with anti-PD-1 & anti-CTLA4 combinational therapy from 01/2016 to 09/2021 at the University Medical Center Freiburg, Dpt. of Dermatology were prospectively included in the discovery cohort (n = 40). A total of 111 patients were identified in clinical records. The remaining (n = 71) patients were retrospectively evaluated in the validation cohort (see Additional file 1: Fig. S2). All included patients had baseline ALT and AST levels below 2xULN and underwent screening for Hepatitis B and Hepatitis C Virus infection. Evaluation of hepatitis was based on ALT, AST and bilirubin evaluations according to CTCAE 5.0. Other adverse events were identified by retrospective evaluation of clinical records. Patients with hepatitis of other etiology were subsequently excluded from the analysis, this affected 1 patient in the discovery cohort was excluded from analysis due to alternative cause of hepatitis (acute HEV infection). 4 patients in the validation cohort were excluded from analysis due to untraceable clinical data and lost to follow-up after therapy initiation. Tumor response was evaluated by radiographic evaluation as per clinical pathways 9–12 weeks from commencement of treatment. Progression (PD) was defined by radiographic disease progression or clinically unequivocal rapid disease progression necessitating cessation of ICB treatment. Tumor regression was determined by radiographic total (CR) or partial (PR) regression of tumor sites. Stable disease (SD) was defined by unchanged radiographic diagnosis. Patients without radiographic evaluation were noted not evaluable (NE). Objective response rate (ORR) was calculated as CR + PR/(total patients-NE); Disease control rate (DCR) was calculated as CR + PR + SD/(total patients–NE). Tumor progression-free survival (PFS) was determined from therapy initiation until the date of tumor progression. Patients that switched therapy before tumor progression were censored at time of therapy switch.

### Human samples

For patients in the discovery cohort, baseline blood was obtained on the day of therapy initiation. Plasma was isolated from EDTA tubes after 10 min of centrifugation at 1000*g* and stored at − 20 °C until use. PBMCs were isolated by density gradient centrifugation and stored at -80C until use. For patients in validation cohort that did not have serology results for CMV, EBV and HSV prior to this study, leftover serum was used for identification of IgG positivity. Leftover serum was from the screening for HBV, HCV and HIV serology before therapy initiation during routine clinical management at the Institute of Virology, University Medical Center Freiburg.

### Ex vivo flow cytometry

Cells were thawed and counted. 1–2*10E6 cells were used for flow cytometry. Surface staining was performed in a total volume of 50 μl antibody master mix at RT for 15 min and washed twice before acquiring on BD LSR Fortessa. For intracellular staining, cells were permeabilized with FoxP3/Transcription Factor Staining Buffer Set (eBioscience) on ice for 30 min and washed twice with FoxP3 permeabilization buffer (eBioscience), followed by intracellular staining in a total volume of 50 μl antibody master mix on ice for 30 min. Cells were fixed with 2% PFA until measurement. Samples were then acquired and recorded on BD LSRFortessa™. For gating strategies see Additional file 1: Fig. S5.

### Statistical analysis

Statistical analysis was performed with Graphpad version 9.0. As indicated in figure legends, data were analyzed using two-tailed Mann–Whitney test, two-tailed chi-square test, Fisher’s exact test, Kruskal–Wallis test, log-rank survival analysis, receiver-operator characteristic (ROC) analysis or pairwise Pearson correlation.

## Results

### High incidence of hepatitis after PD-1 & CTLA-4 combination checkpoint therapy in melanoma

**Fig. 1:**
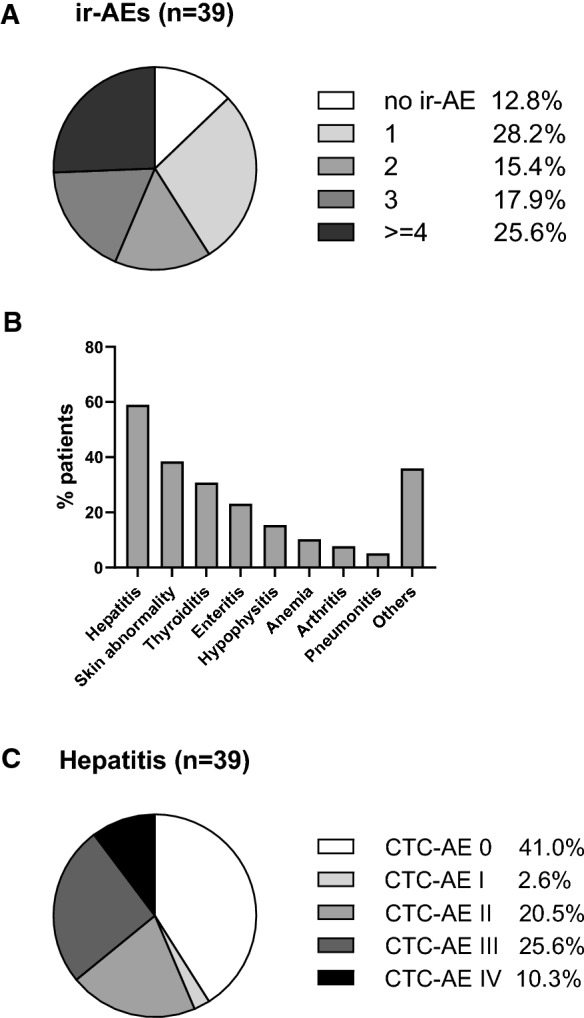
Incidence of immune-related adverse events (irAE) in the first 100 days after initiation of therapy. **A** Pie chart depicting the percentage of patients who developed single or multiple types of irAE in the first 100d after therapy initiation. **B** Bar plot depicting the percentage of patients who developed certain types of adverse events as indicated. **C** Pie chart depicting the percentage of patients who escaped hepatitis or developed hepatitis of indicated severity in the first 100 days post therapy

We first evaluated the incidence of hepatitis and other immune-related adverse events (irAE) in a prospectively recruited discovery cohort of n = 39 patients after initiation of anti-PD-1 & anti-CTLA4 therapy due to advanced melanoma over a period of 100 days. 87.2% of patients (34/39) developed one or more irAE (Fig. 1A). While 28.2% of patients developed a single adverse event, 15.4% developed 2 types of adverse events and 17.9% and 25.6% of patients developed 3 or more types of adverse events. Specifically, we observed a high incidence of hepatitis irAE (59%) in our cohort (Fig. 1B). While 2.6% of patients developed mild hepatitis (grade 1), 20.5% developed moderate hepatitis (grade 2) and 25.6% and 10.3% developed severe grade 3 and 4 hepatitis, respectively, requiring immunosuppressive therapy and treatment pause or discontinuation (Fig. 1C). There was no grade 5 toxicity. In sum, we observed a relatively high rate of hepatitis incidence in the first 100 days after anti-PD-1 & CTLA-4 treatment initiation for advanced melanoma.

### Hepatitis onset is associated with the development of additional irAE and age but not gender or treatment response

**Fig. 2:**
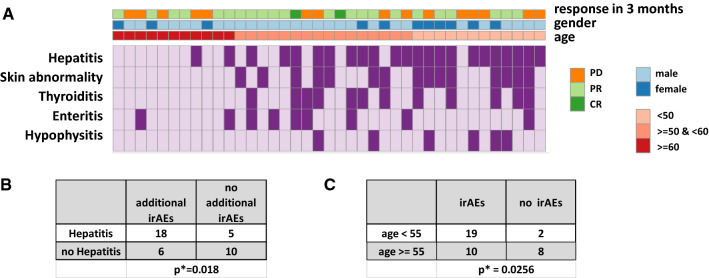
Hepatic irAE frequently co-occur with other irAE and are associated with age. **A** Heatmap displaying the distribution of Hepatitis, skin abnormality, thyroiditis, enteritis and hypophysitis across tumor response, gender and age. Events are highlighted in dark purple. **B** Table showing the absolute number of patients who developed thyroiditis, hypophysitis, skin abnormality or enteritis grouped by the incidence of hepatitis. **C** Table showing number of patients with and without top 5 ir-AEs across age groups. Statistics were determined by Fisher’s exact test in **B** and chi square test in **C**

We next aimed to understand if development of hepatitis was associated with the development of other irAE, response to treatment, gender or age. Our discovery cohort consisted of patients with an age distribution between 19–73 years, a male dominance (71.8%) and ORR of 61.5% after 3 months, reflecting the real-life setting in our tertiary clinical centre (Additional file 1: Fig. S1). As shown in Fig. 2A, there was no association of hepatitis incidence with the oncologic response after three months (p = 0.7397) nor gender (p > 0.99) (Additional file 1: Table S1). Patients with hepatitis had significantly higher co-incidence of skin, thyroid, gastrointestinal or hypophysal irAE (p = 0.018, Fig. 2B). Development of irAE was significantly associated with age, with patients over 55 years old exhibiting higher incidence for top five frequent irAE compared to younger patients under 55 (Fig. 2C). These data indicate that age might be a determining correlate of the onset of hepatitis and other irAE in our cohort.

### Younger age is associated with incidence and severity of hepatic irAE in the discovery cohort

**Fig. 3:**
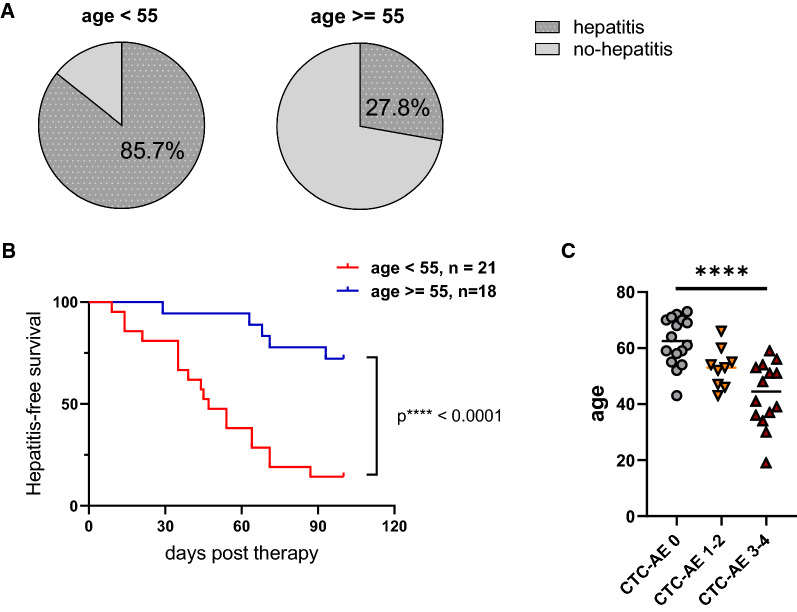
Age is associated with higher frequency, earlier onset and increased severity of therapy-induced hepatitis. **A** Pie charts depicting the percentage of patients with therapy-induced hepatitis. **B** Kaplan–Meier analysis of hepatitis events in young patients (< 55, n = 21) and aged patients (≥ 55, n = 18). **C** Dot plot describing the age of patients without hepatitis, or who developed mild hepatitis (CTC-AE 1–2) or severe hepatitis (CTC-AE 3–4) Statistics were performed by log-rank test (**B**) and Kruskal–Wallis test (**C**)

We next analyzed the connection between age and hepatitis incidence in more detail. Of note, 85.7% of patients under age 55 had hepatitis during the first 100 days of therapy, while only 27.8% of patients older than 55 years had hepatitis during our observation period (Fig. 3A). The significantly higher incidence of hepatitis in younger patients was also connected to hepatitis-free survival, an earlier onset and higher severity of hepatitis in younger patients (Fig. 3B, C) in the discovery cohort.

### Younger age is associated with incidence and severity of hepatic irAE in the validation cohort

**Fig. 4:**
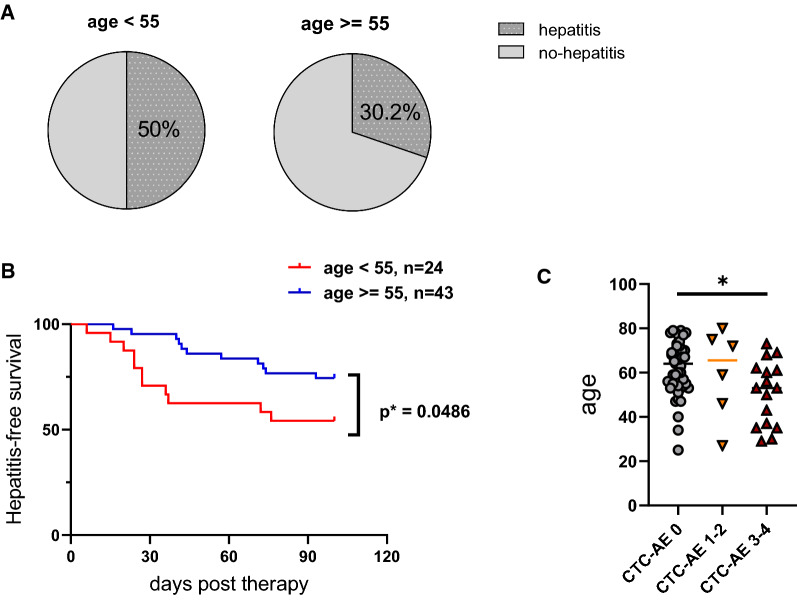
Validation of the age association with Immune-checkpoint-hepatitis in another cohort of 67 patients (validation cohort). **A** Pie charts depicting the percentage of patients with therapy-induced hepatitis. **B** Kaplan–Meier analysis of hepatitis events in young patients (< 55, n = 24) and aged patients (≥ 55, n = 43). **C** Dot plot describing the age of patients without hepatitis, or who developed mild hepatitis(CTC-AE 1–2) or severe hepatitis (CTC-AE 3–4) Statistics were performed by log-rank test (**B**) and Kruskal–Wallis test (**C**)

To exclude the possibility that patient characteristics other than age might have dictated the association with irAE in our discovery cohort, we sought to validate these findings in a retrospective analysis of all patients treated at our tertiary center during the recruitment period to address recruitment bias. The validation cohort consisted of n = 67 patients (see methods section for inclusion/exclusion criteria and Additional file 1: Fig. S2). We noted several differences in the composition of the validation cohort, namely reduced incidence of irAE, including hepatitis (Additional file 1: Table S2), potentially connected to a shift towards older age (median age 54 years vs. 60 years in discovery and validation cohort, respectively), reduced ORR (61.5% vs. 28.6%, respectively) while gender distribution was similar (Additional file 1: Table S2). Importantly, however, we also observed a significant association of younger age with hepatitis-free survival, severity and earlier onset in the validation cohort (Fig. 4). Together, these data support age as a validated risk factor for the development of hepatitis in the first 100 days after anti-PD-1 & anti-CTLA4 therapy.

### Higher baseline liver function tests in patients that develop hepatitis

Interestingly, patients who developed hepatitis within 100 days from therapy initiation had small however significantly higher liver transaminase levels at baseline (Additional file 1: Fig. S3A). This association was observed despite the majority of patients (n = 92, 86.8%) having transaminase levels within the normal range. ROC analysis showed a slight predictive role of both baseline AST and ALT values for ICB-induced hepatitis (Additional file 1: Fig. S3B). Our study also included few patients (n = 14, 13.2%) that had elevated liver transaminases already at baseline (up to 2xULN). However, these few patients with elevated liver transaminases (above the ULN) at baseline did not show a significantly higher incidence of developing further hepatitis during therapy in comparison to those with normal liver transaminase levels (Additional file 1: Fig. S3C). In sum, these data suggest that preexisting mild liver inflammation can be associated with the onset of ICB hepatitis.

### Baseline herpes virus serology is not significantly linked to hepatitis onset

**Fig. 5:**
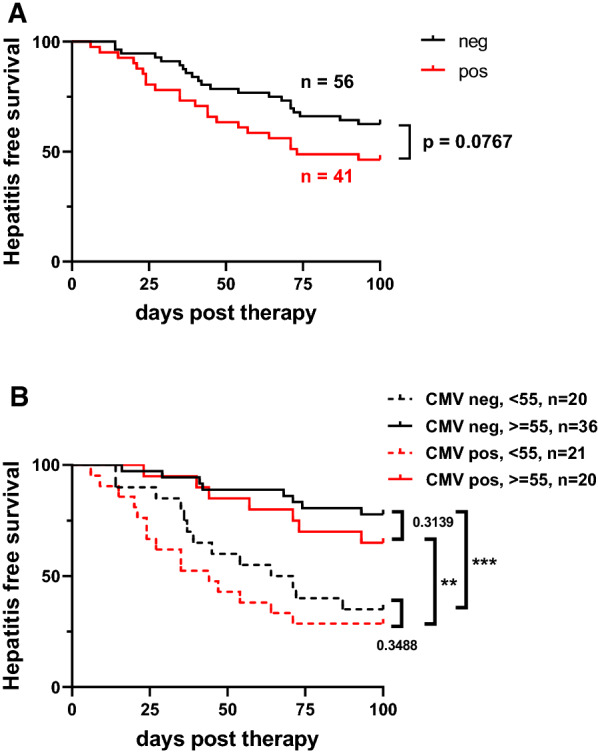
Incidence of Immune-checkpoint-hepatitis does not correlate with CMV serostatus. **A** Kaplan-Meier analysis of hepatitis events in patients grouped by IgG positivity against CMV. **B** Kaplan-Meier analysis of hepatitis events in young (< 55) and aged (≥ 55) patients with either negative or positive CMV IgG status

The immune system is exposed to multiple antigens over time and immunological changes associated with herpes virus infections are connected to age [16]. A previous report described baseline antiviral T cell immunity to herpes virus infections as a potential driver of hepatic irAE [17]. We wondered if this association would explain the age-associated differences in the incidence of hepatitis in our cohort, since higher hepatitis incidence was observed in younger patients who would be predicted to have lower immune memory to herpes virus infections. Herpes virus serology for CMV, EBV and HSV was determined and analyzed with respect to hepatitis incidence. However, we did not observe an association of herpes virus serology with hepatitis-free survival in our observation and validation cohorts (Additional file 1: Fig. S4). Interestingly, in pooled analysis of both cohorts, positive serology for CMV at baseline showed a non-significant trend (p = 0.0767) towards lower hepatitis-free survival (Fig. 5A). CMV status was not connected to baseline liver transaminases (Additional file 1: Fig. S3D). Since the effect of CMV positivity on hepatitis incidence was not significant, we wondered if it might be masked by different age groups. Subgroup analysis according to CMV serostatus positivity and age (cutoff 55 years) showed however again only non-significant trends towards lower hepatitis free incidence (Fig. 5B). In sum, we did not observe a significant difference in hepatitis-free survival connected to herpes-virus immunity in our cohorts.

### T cell immunity is altered in older patients but not predictive for hepatitis incidence

**Fig. 6:**
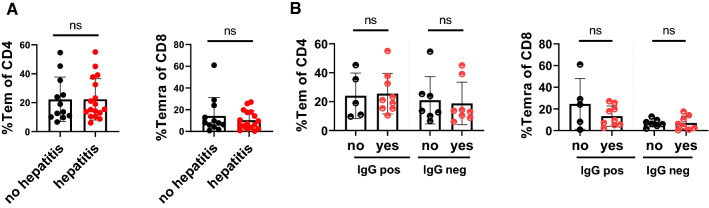
Frequency of effector memory T cells is not associated with ICB-hepatitis. In a sub-cohort (n = 29), T cell phenotypes at baseline were determined. For **A** %Tem(CCR7-CD45RA-) of CD4 (left) and Temra (CCR7-CD45RA + CD27−) of CD8 (right), no difference was observed between patients with or without Immune-checkpoint-hepatitis. **B** Patients were based on CMV-IgG serostatus (positive/negative), no difference of %Tem CD4 or %Temra CD8 was observed in patients with or without Immune-checkpoint hepatitis

Preexisting T cell memory is discussed to underlie immune-mediated toxicity after checkpoint therapy and would fit to a model where the immune checkpoints targeted contribute to attenuation of autoimmunity in a physiological setting. We therefore performed a detailed analysis of CD8 and CD4 T cell subsets in the discovery cohort and analyzed potential associations of these immune populations (Fig. 6, Additional file 1: Figs. 5 and 6) with age and the incidence of hepatitis. As expected [16], we observed a reduction of naïve CD8+ T cells in older patients while memory populations expanded (Additional file 1: Figs. S6A and SB). However, we observed no difference between these age-associated T cell populations and viral hepatitis (Additional file 1: Fig. S6C). An association of an expanded CD4+ effector memory T cell (TEM) population with ICB-hepatitis was reported earlier [17]. However, we did not observe different frequencies of CD4 + TEM or CD8+ TEMRA cells in patients who subsequently developed hepatitis after anti-PD-1 & anti-CTLA-4 therapy (Fig. 6A), and there was no difference in the T cell subset distribution between patients which did or did not develop hepatitis according to CMV serology status (Fig. 6B). These data indicate that differences in baseline T cell differentiation subsets are not directly linked to the onset of hepatitis after anti-PD-1 & anti-CTLA-4 checkpoint therapy.

### Age as a potential predictor for therapy-induced hepatitis but not for tumor response

**Fig. 7:**
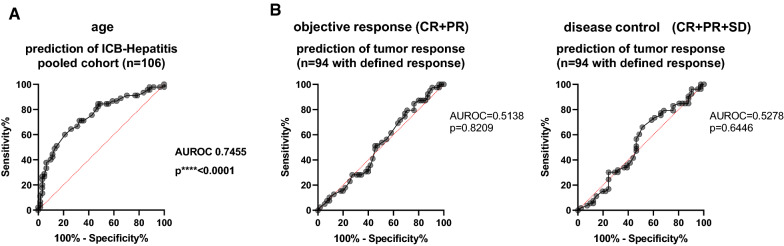
Age is a predictor for anti-PD-1/anti-CTLA4 therapy associated Immune-checkpoint hepatitis. **A** ROC analysis using age as discriminator for therapy induced hepatitis the pooled cohort (n = 106). **B** ROC analysis using age as discriminator for tumor response in 3 months in the total cohort of n = 94 patients whose response was assessable. Treatment response was either defined by objective response (CR + PR) (left) or disease control (CR + PR + SD) (right)

We next tested other possible clinical, oncological (BRAF/NRAS status, stage, LDH levels, presence of metastasis), virological or hepatological characteristics of our patient cohorts that might contribute to ICB-hepatitis during dural checkpoint therapy in the pooled cohort (Additional file 1: Table S3). In addition to age, AST and ALT levels, interestingly, this analysis also indicated a significant association of anti-HBs positivity with ICB-hepatitis, while anti-HBc or HBsAg were not significantly associated, in line with a status post HBV vaccination. We interpreted this finding as an age-dependent cohort effect due to wider introduction of HBV vaccination in younger patients. Since age was the most significant variable associated with ICB-hepatitis, we next tested if age could be used as a predictor of the development of ICB-Hepatitis using receiver operating characteristic (ROC) curve analysis. Indeed, age was a relatively reliable discriminator of patients who developed or escaped therapy-induced hepatitis (n = 106, AUROC = 0.7455, p < 0.0001) (Fig. 7A). Specifically, for an age cut-off of 55 years, ROC analysis indicated specificity of 66.67% and sensitivity of 75%respectively in the pooled analysis). We did not observe a similar predictive role for herpes virus serostatus (data not shown). Interestingly, this predictive function of age was not observed with respect to tumor response in the comparable time frame (Fig. 7B). Moreover, presence of any IRAE (including non-hepatitis irAE) was also not associated with improved survival in our cohort (Additional file 1: Fig. S7). This data suggests that the immunological mechanisms behind successful anti-tumor responses and hepatic adverse events are not necessarily connected. In sum, our data highlights age as a predictor of ICB-hepatitis.

## Discussion

In this work we analyzed baseline clinical, immune and virological variables as potential predictors of anti-PD-1 & anti-CTLA-4 combination therapy associated ICB-hepatitis in patients with stage III/IV melanoma. We identified age as the major clinical variable associated with the incidence, early onset and severity of immune hepatitis in our prospectively recruited discovery and retrospective validation cohort independent of treatment efficacy. Of note, preexisting antiviral immunity against herpes virus infections did not significantly associate with the incidence of hepatitis. Moreover, differences in effector memory T cell subsets at baseline in our discovery cohort were associated with age but not with the risk for developing ICB-hepatitis. Our data therefore highlights younger age as the major clinical risk factor ICB-hepatitis in combination therapy and does not support close surveillance or prophylactic antiviral treatment strategies based solely on immunological and virological screening.

One of the main barriers for successful anti-PD1 & anti-CTLA4 therapy are severe adverse events occurring in particular during combinational therapy cycles [6]. The efficacy of anti-PD-1 & anti-CTLA blockade is thought to largely depend on the disinhibition of tumor-specific T cell populations controlled by the PD-1 and CTLA4 immune checkpoints for enhanced proliferation and tumor cytotoxicity. However, checkpoint blockade induced T cell activation may not be strictly confined to tumor-reactive repertoires and “off-target” activation can potentially contribute to immune-related adverse events, a concept that is supported by recent studies revealing enriched activated/cytotoxic T cell populations in the tissue site of adverse events [10–12, 17, 18]. In particular, bystander activation of T cells leading to hepatitis can occur independent of antigen recognition [19] in the context of an inflammatory cytokine milieu [20].

Hutchison et al. recently suggested a role of CMV-related T cell immune response in triggering therapy induced hepatitis by demonstrating enrichment of a CMV-associated CD4 TEM population in the periphery of patients who later developed hepatitis [17]. It has to be noted however, that their study did not show direct evidence of CMV presence in the liver in patients tested (CMV immunostaining and PCR negative), despite individual treatment decisions with antivirals as prophylaxis or in addition to immunosuppressive therapy. Our study used a related approach to profile immune responses in a cohort with comparable baseline patient characteristics but did not identify the reported relationship of hepatitis incidence connected to CD4 TEM cells. Further, serological IgG positivity at baseline against CMV, EBV or HSV did also not significantly correlate with hepatitis incidence. We wondered if these discrepant results in our prospectively recruited discovery cohort as well as the validation cohort could be due to differences in the patient cohorts.

Patients with preexisting mild levels of hepatitis could have other mild forms of underlying liver diseases, but potentially also herpes virus-related inflammation. A sub-analysis by Hutchinson et al. who included patients with elevated liver transaminases at baseline, did not find an association of this baseline status with the incidence of hepatic irAE after therapy [17]. Similarly, our cohort included patients with predominantly normal liver function tests at baseline but also potentially mild hepatitis (ALT levels < 2 ULN according to clinical guidelines allowing these mild elevations for ICB therapy). Here, we did not observe a connection between baseline transaminase levels and CMV serostatus. However, patients that developed ICB-hepatitis had mildly higher transaminase levels at baseline in our cohort. This baseline transaminase elevation at the cohort level however occurred frequently below the ULN (Additional file: Fig. S3). Thus, while this observation points to a higher degree of underlying liver inflammation in patients that subsequently develop hepatitis, it also poses a challenge for identifying them based on liver function tests.

In sum, in this work, we could not confirm a clinically relevant role of virus serology or T_EM_ CD4 T cell populations in patients who later developed hepatitis as previously reported. In contrast, our clinical data revealed a strong predisposition of younger patients to develop hepatitis during therapy, while no such link was observed with tumor response. This data also suggests that immunological mechanisms responsible for successful tumor suppression and incidence of immune mediated hepatitis are not necessarily coupled. It is further exemplified by 2 responders (1 reached CR in 3 months and the other in 6 months) in our discovery cohort that were both exempted from any type of adverse events. This disassociation between tumor response and adverse events necessitates further in-depth research to understand the underlying immunological mechanisms accounting for the respective biological events and their relationship to different age groups. Our data shows that younger patients are at higher risk for developing immune-related hepatitis after combination of anti-PD-1 & anti-CTLA4 therapy and should be closely monitored to allow rapid identification and treatment of this side effect when it occurs.

## Conclusions

Taken together, our work highlights younger age but not TEM expansion or herpes virus immunity as a clinically relevant predictive factor for the onset of anti-PD-1 & anti-CTLA4 related immune hepatitis. These findings have implications for the monitoring of patients at risk for developing checkpoint hepatitis during immunotherapy.

## Supplementary Information

**Additional file 1: Fig. S1.** Characteristics of discovery cohort. (A) Binned histogram depicting age distribution of patients (n = 39, min–max [median]: 19–73 [54]). (B) and (C) Pie charts depicting cohort gender distribution and tumor response in the first 3 months. **Fig. S2. **Workflow of patient selection. Melanoma patients treated with anti-PD1 & anti-CTLA4 combination therapy in Dermatology, University Hopsital of Freiburg between 01/2016–09/2021 were identified in clinical records, with n = 40 in the prospective discovery cohort and n = 71 in retrospective validation cohort. Patients with untraceable clinical data after therapy initiation and patients with hepatitis with other etiology in parallel were further excluded. A total of 106 patients were evaluated for irAE, tumor response and other clinical information. N = 96 plasma was used for IgG serostatus detection against CMV/EBV/HSV. N = 29 PBMCs were analyzed for immune populations at baseline. **Fig. S3.** Baseline AST and ALT values are associated with Immune-checkpoint hepatitis. Baseline AST and ALT values are identified from medical records. Slight elevation of baseline AST (A, left) and ALT (A, right) values were observed in patients who developed hepatitis within 100 days after therapy initiation. (B) ROC analysis showed potential role of baseline AST and ALT values in predicting hepatitis incidence. (C) Incidence of hepatitis in patients stratified by baseline liver transaminases levels. P value was determined by Fisher’s exact test. (D) and (E) Sub-cohort analysis of baseline AST, ALT values stratified by CMV IgG serostatus or age. **Fig. S4.** EBV/HSV/CMV IgG serostatus does not correlate with Immune-checkpoint hepatitis. Kaplan–Meier analysis of hepatitis events in patients grouped by IgG positivity against EBV and HSV in pooled cohort (A), observation cohort (B) and validation cohort (C). (D) Incidence of hepatitis in patients stratified by anti-viral IgG serostatus. (E) Sub-cohort analysis on patients with normal baseline liver transaminase levels stratified by CMV serostatus with or without age subgroups. **Fig. S5.** Gating strategies for CD4 and CD8 subpopulations. CD4 and CD8 T cells are gated on the basis of live CD3 + T cells. Naïve CD8 T cells are gated as CCR7 + CD45RA + CD27 + CD8, central memory (Tcm) gated as CCR7 + CD45RA-CD27 + CD8, early effector memory (early Tem) gated as CCR7-CD45RA-CD27 + CD8, late effector memory (late Tem) gated as CCR7-CD45RA-CD27-CD8, Temra gated as CCR7-CD45RA + CD27-CD8. Effector memory CD4 T cells (Tem CD4) are gated as CCR7-CDRA-CD4, as in Hutchinson et al. **Fig. S6**. Age-associated T cell populations are not related to incidence of Hepatitis. Peripheral T cell populations were quantified in 29 melanoma patients before cCBI. (A) Spearman correlation analysis of T cell sub-population distribution with increasing age. Correlation strength and directionality (left: negative correlation, right: positive correlation) is shown for each T cell sub-population as indicated. Correlation significance for each population is denoted by shading (p < 0.01, black, p < 0.05, gray). (B) linear regression between age and %Tn CD8 (left) or %TIGIT + CD8 (right). Patients without hepatitis are represented in black dots, patients with mild (CTC-AE I-II) hepatitis in purple dots and patients with severe (CTC-AE III-IV) hepatitis in blue dots. (C) Comparisons of %Tn CD8 (left) and %TIGIT + CD8 T cells (right) between patients without hepatitis and patients with mild or severe hepatitis. Statistics was performed with Mann–Whitney test. **Fig. S7.** Tumor progression-free survival analysis according to ICB-hepatitis, age and irAE. Kaplan–Meier analysis of tumor progression free survival (PFS) was performed based on (A) incidence of hepatitis, (B) age (cutoff 55 years) and (C) incidence of at least one of the top 5 most frequent (hepatitis, enteritis, thyroiditis, hypophysitis and skin abnormality) irAE in our cohort.

## Data Availability

Data relevant to the study are included in the article or uploaded as online supplemental information. Flow cytometry data is available by the authors via https://flowrepository.org upon reasonable request.

## Abbreviations

cCBI: Combinational checkpoint blockade immunetherapy
irAE: Immune-related adverse events
Tem: Effector memory T cells
Temra: CD45RA positive effector memory T cells
CR: Complete response
PR: Partial response
SD: Stable disease
PD: Progressive diseaseCR
NE: Not evaluable
ORR: Objective response
DCR: Disease control rate
PFS: Tumor progression-free survival
