# Supplementary material for: Incidence and severity of immune-related hepatitis after dual checkpoint therapy is linked to younger age independent of herpes virus immunity
Source: J Transl Med. 2022 Dec 12;20:582. doi: 10.1186/s12967-022-03755-3 (PMC9743538; doi:10.1186/s12967-022-03755-3)
Supplement: Supplementary file 1 — Additional file 1: Fig. S1. Characteristics of discovery cohort. (A) Binned histogram depicting age distribution of patients (n = 39, min–max [median]: 19–73 [54]). (B) and (C) Pie charts depicting cohort gender distribution and tumor response in the first 3 months. Fig. S2. Workflow of patient selection. Melanoma patients treated with anti-PD1 & anti-CTLA4 combination therapy in Dermatology, University Hopsital of Freiburg between 01/2016–09/2021 were identified in clinical records, with n = 40 in the prospective discovery cohort and n = 71 in retrospective validation cohort. Patients with untraceable clinical data after therapy initiation and patients with hepatitis with other etiology in parallel were further excluded. A total of 106 patients were evaluated for irAE, tumor response and other clinical information. N = 96 plasma was used for IgG serostatus detection against CMV/EBV/HSV. N = 29 PBMCs were analyzed for immune populations at baseline. Fig. S3. Baseline AST and ALT values are associated with Immune-checkpoint hepatitis. Baseline AST and ALT values are identified from medical records. Slight elevation of baseline AST (A, left) and ALT (A, right) values were observed in patients who developed hepatitis within 100 days after therapy initiation. (B) ROC analysis showed potential role of baseline AST and ALT values in predicting hepatitis incidence. (C) Incidence of hepatitis in patients stratified by baseline liver transaminases levels. P value was determined by Fisher’s exact test. (D) and (E) Sub-cohort analysis of baseline AST, ALT values stratified by CMV IgG serostatus or age. Fig. S4. EBV/HSV/CMV IgG serostatus does not correlate with Immune-checkpoint hepatitis. Kaplan–Meier analysis of hepatitis events in patients grouped by IgG positivity against EBV and HSV in pooled cohort (A), observation cohort (B) and validation cohort (C). (D) Incidence of hepatitis in patients stratified by anti-viral IgG serostatus. (E) Sub-cohort analysis on patients with [file 12967_2022_3755_MOESM1_ESM.pdf]

s.Fig.1 Characteristics of discovery cohort.

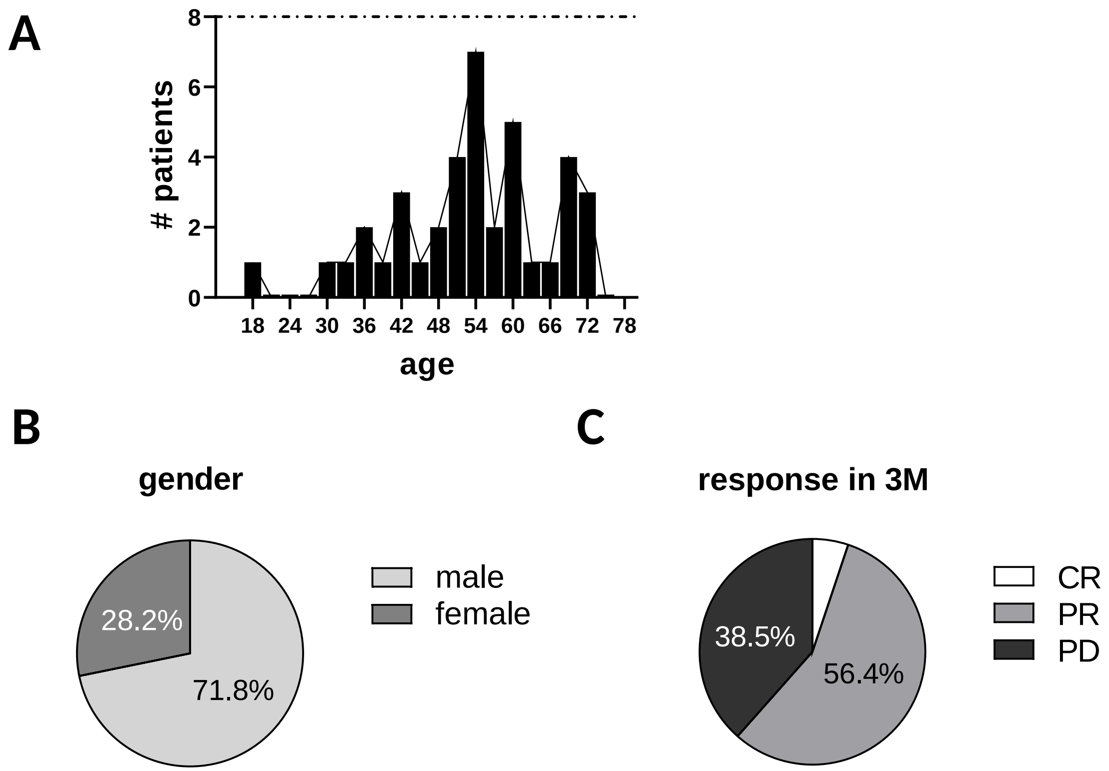

s.Fig.2 Workflow of patient selection

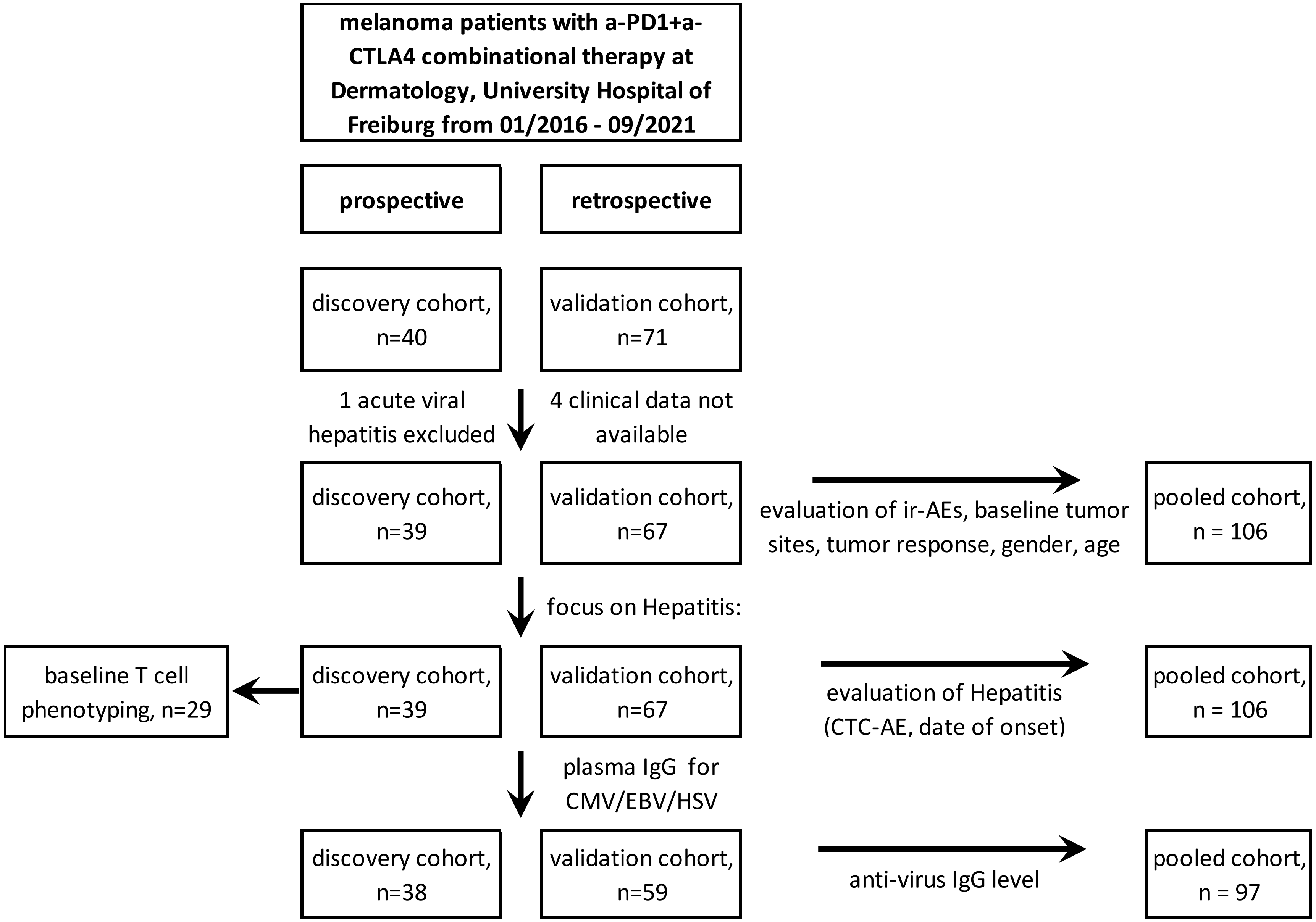

s.Fig.3 Baseline AST and ALT values are associated with therapy-induced hepatitis.

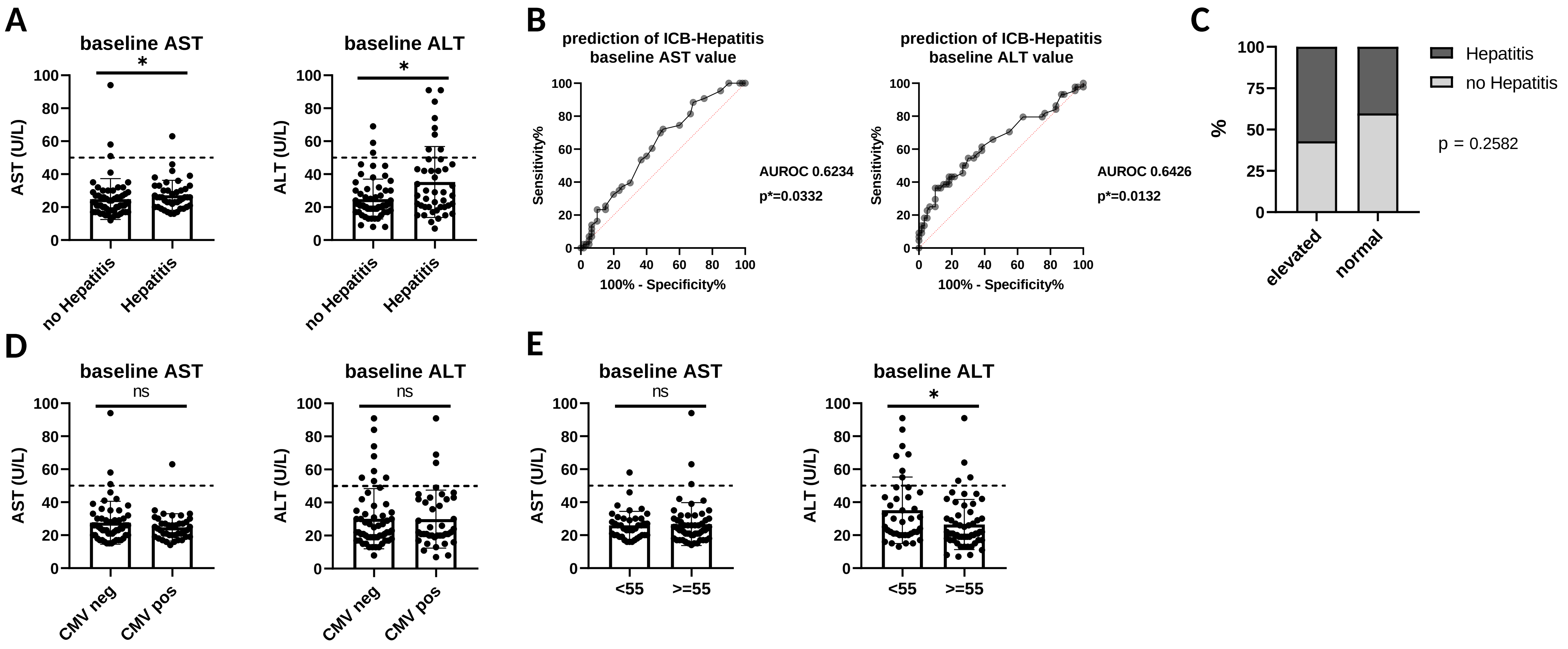

s.Fig.4 EBV/HSV/CMV IgG serostatus does not correlate with Immune-checkpoint hepatitis.

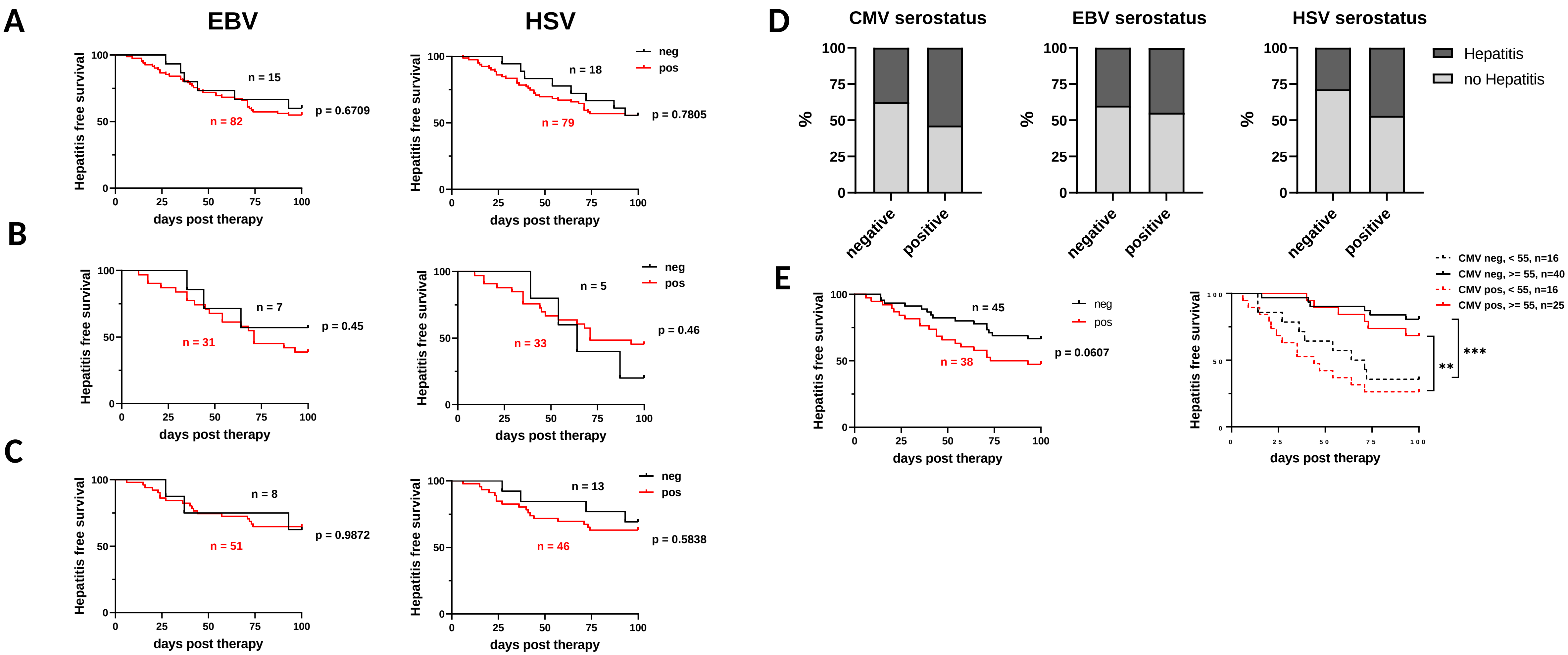

s.Fig.5 Gating strategies for CD4 and CD8 subpopulations.

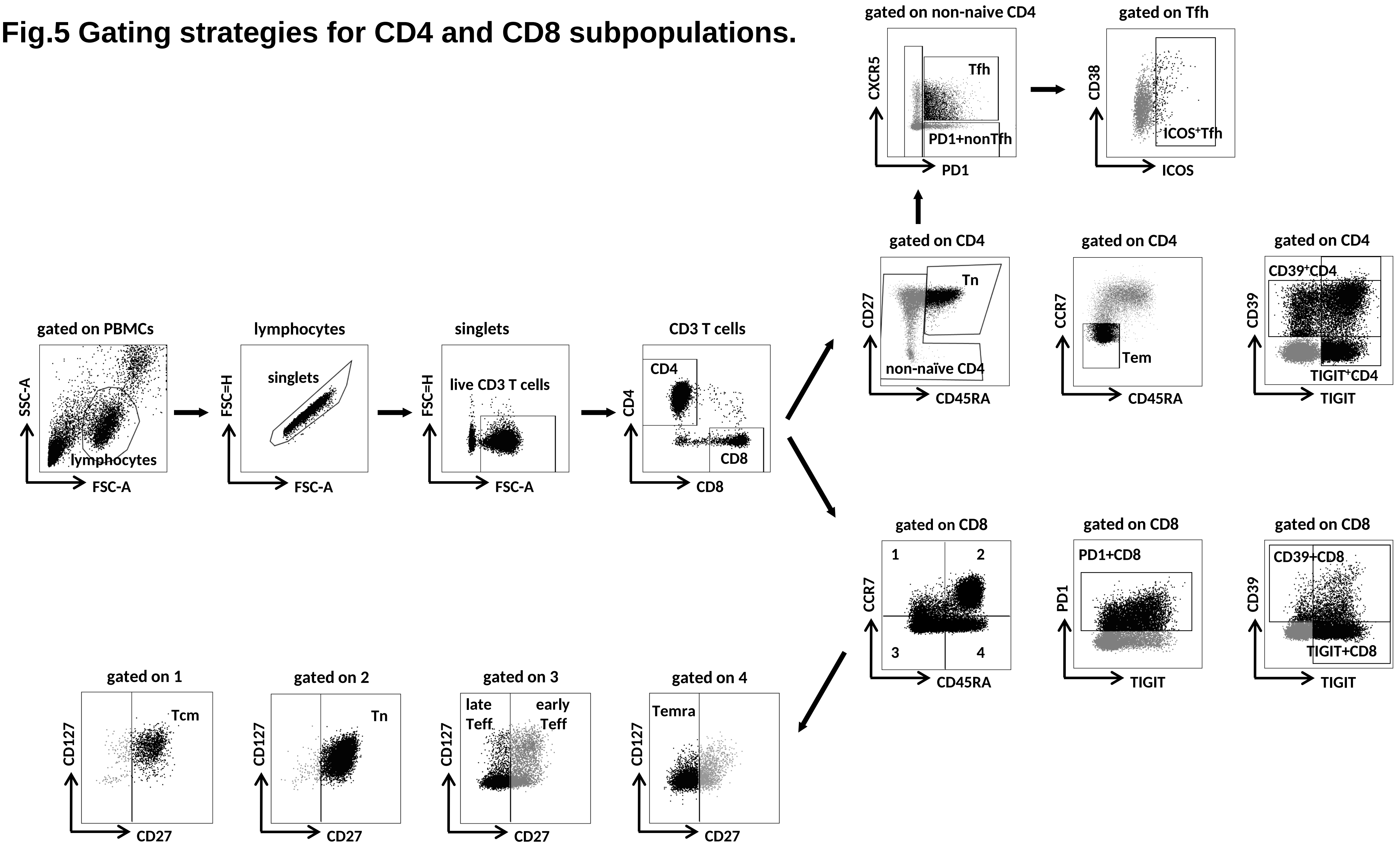

s.Fig.6 Age-associated T cell populations are not related to incidence of Hepatitis

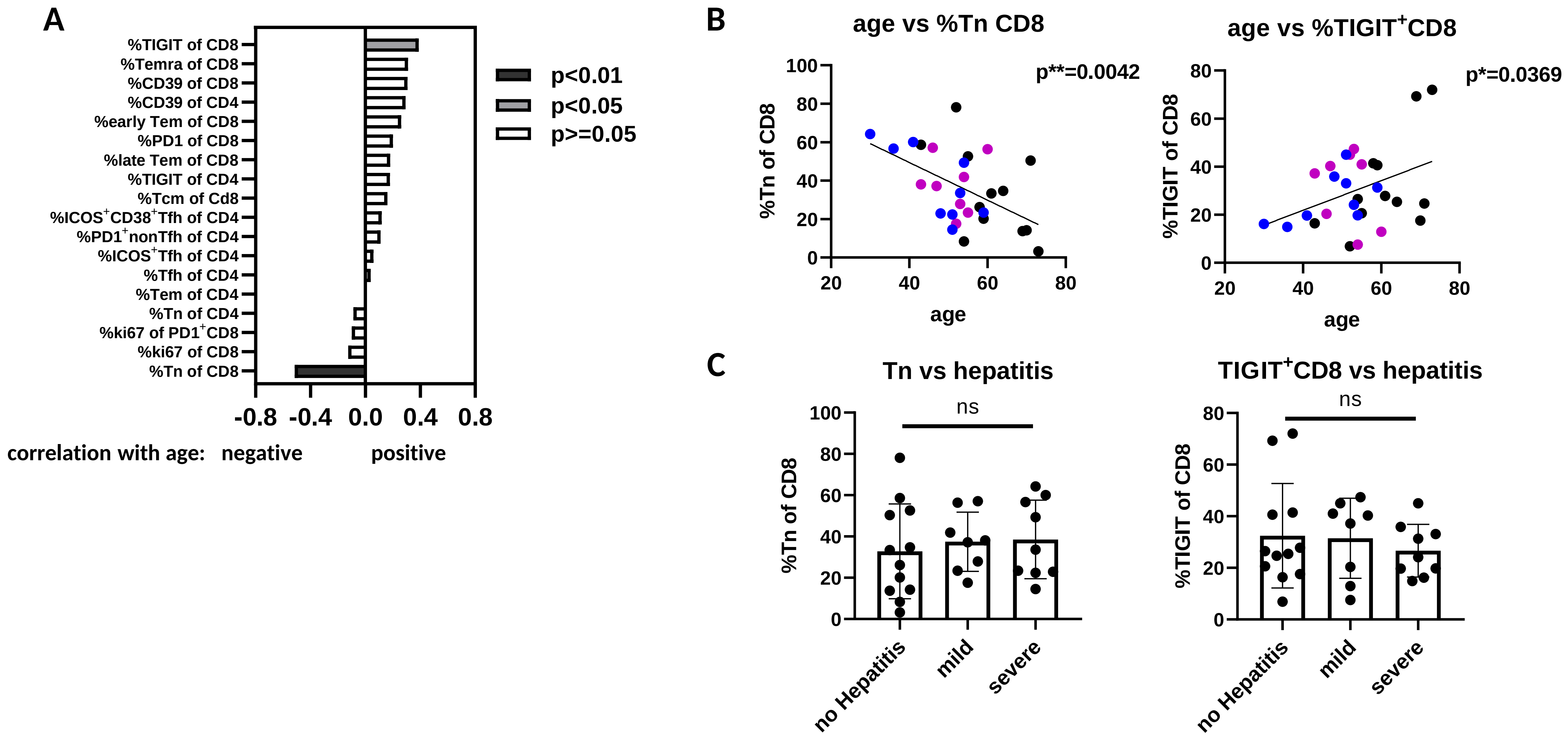

s.Fig.7 Tumor progression-free survival analysis according to ICB-hepatitis, age and irAE

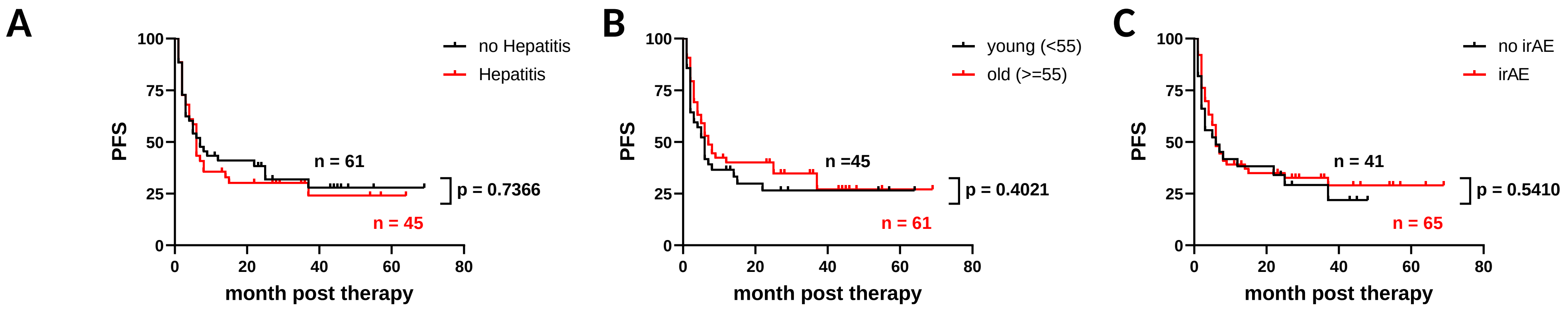

Supplemental Table 1:

	hepatitis	no hepatitis	total
responders(CR+PR+SD)	16	9	25
non-responders(PD)	8	6	14

p=0.7397

	hepatitis	no hepatitis	total
male	16	12	28
female	7	4	11

p>0.99

Contingency table comparing distribution of tumor response and gender in patients with/without hepatitis in discovery cohort.

Supplemental Table 2:

	discovery cohort(n=39)	validation cohort(n=67)	test performed	p value
**age**				
median age	54	60	Mann-Whitney Test (two-tailed)	0.0305
min. age	19	25		
max. age	73	80		
**gender**				
male	28	46	Chi Square Test	0.7343
female	11	21		
**tumor response in 3 months $**				
CR	2	7	Chi Square Test	<0.0001
PR	22	9		
SD	0	14		
PD	15	26		
NE	0	11		
**no. of ir-AEs**				
0	8	29	Chi Square Test	0.0016
1	10	18		
2	6	15		
3	9	4		
>= 4	6	1		
**incidence of Hepatitis**				
no Hepatitis	16	45	Chi Square Test	0.0086
Hepatitis	23	22		
**severity of Hepatitis**				
CTC-AE I	1	1	Chi Square Test	0.8369
CTC-AE II	8	5		
CTC-AE III	10	12		
CTC-AE IV	4	4		

Clinical information of patients included in the discovery and validation cohort.

$ CR: complete response, PR: partial response, PD: progression disease, SD: stable disease,

NE: not evaluable. Comparison excluded patients with NE response.

Supplemental Table 3

	No. of patients evaluated	no Hepatitis, n=61	Hepatitis, n=45	test performed	p value
**age**					
median age	n=106	64	52	Mann-Whitney Test (two-tailed)	**<0.0001**
min. age		25	19		
max. age		79	80		
**gender**					
male	n=106	46	28	Chi Square Test	0.1438
female		15	17		
**tumor response in 3 months $**					
CR	n=94	4	4	Chi Square Test	0.1482
PR		13	18		
SD or MR		11	3		
PD		23	18		
NE		10	2		
**CMV IgG status**					
positive	n=97	24	22	Chi Square Test	0.5104
negative		30	21		
**AST**					
median(95% CI)	n=101	21.5(20-26)	26(23-29)	Mann-Whitney Test (two-tailed)	**0.0326**
**ALT**					
median(95% CI)	n=102	21(19-24)	28(22-42)	Mann-Whitney Test (two-tailed)	**0.0128**
**liver metatstasis**					
no	n=106	46	31	Chi Square Test	0.4567
yes		15	14		
**no. of non-Hepatitis irAE ***					
0	n=106	41	15	Chi Square Test	**0.0003**
1		17	15		
2		3	11		
3		0	4		
4		0	0		
**BRAF $**					
other mutations	n=101	1	4	Chi Square Test	0.1784
V600 mutation		24	16		
wt		35	21		
unknown		1	4		
**NRAS $**					
other mutations	n=91	2	1	Chi Square Test	0.7508
Q61 mutation		15	14		
wt		35	24		
unknown		9	6		
**stage**					
III	n=106	3	2	Fisher's Exact Test (two-tailed)	>0.9999
IV		58	43		
**LDH level**					
normal	n=93	26	23	Chi Square Test	0.5587
elevated		26	18		
**HBsAg**					
negative	n=99	55	43	Fisher's Exact Test (two-tailed)	>0.9999
positive		1	0		
**anti-HBc antibody**					
negative	n=99	53	39	Fisher's Exact Test (two-tailed)	0.4638
positive		3	4		
**anti-HBs antibody**					
negative	n=99	44	26	Chi Square Test	**0.0497**
positive		12	17		
**anti-HCV antibody**					
negative	n=98	55	43	Fisher's Exact Test (two-tailed)	>0.9999
positive		0	0		
**anti-HIV antibody**					
negative	n=98	55	43	Fisher's Exact Test (two-tailed)	>0.9999
positive		0	0		

f patients who developed hepatitis or not in pooled cohort
* only included skin abnormality, thyroiditis, enteritis and hypophysitis
$ Comparison excluded patients with NE response or unknown status
